# Exploring the association between whole blood Omega-3 Index, DHA, EPA, DHA, AA and n-6 DPA, and depression and self-esteem in adolescents of lower general secondary education

**DOI:** 10.1007/s00394-018-1667-4

**Published:** 2018-03-16

**Authors:** I. S. M. van der Wurff, C. von Schacky, T. Bergeland, R. Leontjevas, M. P. Zeegers, P. A. Kirschner, R. H. M. de Groot

**Affiliations:** 1 Welten Institute, Research Centre for Learning, Teaching, and Technology, Open University of the Netherlands, Valkenburgerweg 177, P.O. Box 2960, 6419 AT Heerlen, The Netherlands; 2 Omegametrix, 82 152 Martinsried, Germany; 3 Preventive Cardiology, Medical Clinic and Poli-Clinic I, Ludwig Maximilians-University Munich, Munich, Germany; 4 Aker BioMarine Antarctic AS, 1327 Lysaker, Norway; 5 Faculty of Psychology and Educational Sciences, Open University of the Netherlands, 6419 AT Heerlen, The Netherlands; 6 Nutrition and Translational Research in Metabolism (School NUTRIM), Maastricht University, 6200 MD Maastricht, The Netherlands; 7 Care and Public Health Research Institute (School CAPHRI), Maastricht University, 6200 MD Maastricht, The Netherlands; 8 University of Oulu, Oulu, Finland

**Keywords:** Adolescents, Depressed mood, Long-chain polyunsaturated fatty acid, Self-esteem, Omega-3 Index, Healthy youth, High schoolers, Brain functioning

## Abstract

**Purpose:**

Depression is common in adolescents and long-chain polyunsaturated fatty acids (LCPUFA) are suggested to be associated with depression. However, research in adolescents is limited. Furthermore, self-esteem has never been studied in relation to LCPUFA. The objective here was to determine associations of depression and self-esteem with eicosapentaenoic acid (EPA), docosahexaenoic acid (DHA), Omega-3 Index (O3I), n-6 docosapentaenoic acid (n-6 DPA, also called Osbond acid, ObA), n-3 docosapentaenoic acid (DPA), and arachidonic acid (AA) concentrations in blood of adolescents attending lower general secondary education (LGSE).

**Methods:**

Baseline cross-sectional data from a krill oil supplementation trial in adolescents attending LGSE with an O3I ≤ 5% were analysed using regression models built with the BayesFactor package in R. Fatty acids and O3I were determined in blood. Participants filled out the Centre for Epidemiologic Studies Depression (CES-D) scale and the Rosenberg Self-Esteem scale (RSE).

**Results:**

Scores indicative of depression (CES-D ≥ 16) were found in 29.4% of the respondents. Of all fatty acids, we found extreme evidence [Bayes factor (BF) > 100] for a weak negative association between ObA and depression score [− 0.16; 95% credible interval (CI) − 0.28 to − 0.04; BF_10_ = 245], and substantial evidence for a weak positive association between ObA and self-esteem score (0.09; 95% CI, − 0.03 to 0.20; BF_10_ = 4). When all fatty acids were put in one model as predictors of CES-D or RSE, all of the 95% CI contained 0, i.e., no significant association.

**Conclusion:**

No evidence was found for associations of DHA, EPA and O3I with depression or self-esteem scores in LGSE adolescents with O3I ≤ 5%. The associations of higher ObA status with lower depression and higher self-esteem scores warrant more research.

## Introduction

Depression affects approximately 350 million people worldwide and is especially common in adolescence [1, 2]. It has been suggested that 14–25% of adolescents will experience at least one depressive episode before the age of 18 [3]. Adolescent depression has been associated with many adverse short- and long-term outcomes such as poor social relationships, lower concentration, lower school performance, negative physical and mental health outcome, and increased risk for adult depression [4–7]. Subthreshold depression (i.e., depressive symptoms are present but criteria for a major depression are not met) is also common, with a life-time prevalence up to age 17 varying between 5.3 and 12% [8]. Subthreshold depression has also been related to poorer quality of life, socio-emotional dysfunction, and increased risk for the development of major depression [9, 10].

Depression is a complex and heterogeneous disorder and its aetiology is not completely understood. Many factors have been related to the commencement of depression: heritability, childhood adversity, acute stressful live events, and chronic stress among others [11–15]. Depression can lead to changes in brain structures, production of proinflammatory factors, neuroinflammation, alteration of the immune system, dysfunction and elevation of homocysteine levels, blood flow abnormalities, and a decrease in glucose metabolism [16–21].

Long-chain polyunsaturated fatty acids (LCPUFA) have been suggested to be associated with depression. LCPUFA are important constituents of all cell membranes and are involved in many aspects of brain functioning such as neuronal membrane fluidity, brain blood flow, signal transduction and neurotransmission [22, 23]. Moreover, LCPUFA have been suggested to modulate neuroendocrine factors, have anti-inflammatory properties and may in this way help counteract or reduce the neurobiological changes associated with depression [24]. Multiple observational studies have shown that blood and brain eicosapentaenoic acid (EPA, C20:5n-3) and docosahexaenoic acid (DHA, C22:6n-3) concentrations are lower in people with a depression compared to non-depressed controls [25–28]. Moreover, a number of intervention trials have shown a positive effect of LCPUFA supplementation on depression [29–32], but not all [33, 34].

Studies in adolescents either focus on adolescents in treatment for depression [35, 36], on dietary intake of LCPUFA [37, 38], or on adolescents and adults together [39]. There is only one study, to the best of our knowledge, which investigates LCPUFA status and depressive symptoms in adolescents from the general population.

Mamalakis et al. showed a negative association between EPA measured in adipose tissue and depression score measured by the Centre for Epidemiologic Studies Depression (CES-D) scale after correction for adiponectin, indicating that a lower EPA status was associated with more depression. Furthermore, they showed a positive association between dihomo-gamma linolenic acid (DGLA, C20:3n-6) in adipose tissue and depression score measured with the Beck Depression Inventory, again after correction for adiponectin, indicating that a lower DGLA status is associated with less depression. Last, depression occurs more often in adults and adolescents with a lower socioeconomic status [40, 41]. However, the association between the Omega-3 Index (O3I) measured in blood and depression in adolescents with a lower education level has, to our knowledge, not yet been studied.

Thus, little is known about the association between omega-3 fatty acids and depression in healthy adolescents of a lower educational level. We investigated the association between the O3I, measured in blood and depression in a sample of second year students of lower general secondary education (LGSE). The O3I is the amount of EPA + DHA expressed as a percentage of total identified fatty acids after response factor correction and a correction for the fact that whole blood was used instead of the normally used erythrocytes. We expected that a higher O3I would be associated with less depressive feelings. Second, associations between DHA, EPA, docosapentaenoic acid (DPA, 22:5n-3), arachidonic acid (AA, 20:4n-6), and Osbond acid (ObA also called n-6 docosapentaenoic acid, 22:5n-6) measured in blood and depression were explored. We focussed on AA, DHA and EPA because they are implicated to play a role in mental health [42], whereas ObA is considered to be a functional shortage indicator of DHA [43] and DPA as the major intermediate between DHA and EPA. Therefore, it was expected that higher DHA, EPA and DPA were associated with lower depression scores, and that higher AA and ObA were associated with higher depression scores in adolescents. Furthermore, exploratively, we estimated the associations of the fatty acids with self-esteem. Self-esteem is a core construct of mental health as it represents a person’s overall evaluation of his or her own worth [44]. Low self-esteem has been associated with poor health behaviour and many forms of mental illness, including an association between low self-esteem and major depression in adolescents [44–46] and even negative long-term physical and mental health outcomes [45, 47]. However, to our knowledge, studies are lacking that explore an association between fatty acids and self-esteem.

## Methods

This study used baseline data from a large double-blind, randomised, placebo controlled intervention study (Food2Learn) to study the effect of 1 year of krill oil supplementation on cognitive performance, academic achievement, and mental well-being of students in the second year of LGSE. Full details about the cohort and measurements have been reported previously [48]. Food2Learn was approved by the Medical Ethical Committee of Atrium-Orbis-Zuyd Hospital Heerlen, the Netherlands (NL45803.096.13). Each participant as well as parent(s) and/or guardian(s) provided written informed consent for participation in the study. Food2Learn is registered at The Netherlands Trial Register (NTR4082) and at Clinicaltrials.gov (NCT02240264).

### Data collection

Students of 17 schools offering LGSE in the Netherlands participated in Food2Learn. In the Netherlands, secondary education is divided into three levels: pre-university, higher general and lower general secondary education (LGSE). Approximately 38% of all adolescents in the Netherlands attend LGSE, which is again subdivided into four levels; for the current study, students from the highest sublevel, the theoretical learning pathway (TLP), were recruited. Approximately 40% of students attending LGSE are in the TLP [49]. We approached students in the second year of the LGSE-TLP, who are between 13 and 15 years of age. LGSE-TLP students at participating schools were approached in a classroom setting and the study was explained orally by a research assistant who also used video material to support the presentation. Students then received an information letter and were asked to discuss their participation with their parent(s) and/or guardian(s). If students wanted to participate, they had to hand in an informed consent form signed by both themselves and their parent(s) and/or guardian(s). After informed consent was received the student received a finger prick to determine the O3I. Only students with a low O3I (defined as O3I ≤ 5%) could participate in the study as this was a selection criterion of the main Food2Learn study, as it is to be expected that an effect of supplementation is more likely in those with a low baseline O3I. Thus, participants with an O3I > 5% were not included in the study. At baseline, participants filled out a number of questionnaires to determine mood status and self-esteem, and to collect information with respect to a number of covariates.

### Blood analyses

A finger prick was administered by a trained researcher or research assistant with an automated one-time-use lancet; blood was collected on filtre paper specially prepared with a proprietary antioxidant. Blood acid compositions were analysed according to the HS-Omega-3 Index^®^ methodology as described previously [50, 51]. Fatty acid methyl esters were generated by acid transesterification and analysed by gas chromatography using hydrogen as carrier gas. Fatty acids were identified by comparison with a standard mixture of fatty acids (GLC-727, Nuchek Prep, Elysian, Minnesota, USA). Results are given as the O3I, which is EPA + DHA expressed as a percentage of total identified fatty acids after response factor correction and a correction for the fact that whole blood was used instead of erythrocytes [50]. Typically, the coefficient of variation for EPA plus DHA is 5%. Analyses are quality-controlled according to DIN ISO. Furthermore, 26 other fatty acids were determined. We focussed on AA, DHA, EPA, ObA, and DPA. The O3I has been related to mental health in earlier studies investigating depression, bipolar disorder, and schizophrenia [52–55].

### Questionnaires

#### Centre for Epidemiologic Studies Depression scale

Depressive feelings were assessed with the Dutch version of the CES-D scale [56], one of the most commonly used screening tools for depression that has been shown to be able to distinguish between depressed and non-depressed individuals in both clinical and community populations [56]. The questionnaire consists of 20 questions assessing whether six symptoms of depression were experienced by the participant in the last week. The CES-D has shown a high internal reliability in adolescents (*α* = 0.88) [57]. The measured symptoms include depressed mood, guilt/worthlessness, helplessness/hopelessness, psychomotor retardation, loss of appetite, and sleep disturbance. For each question, the participants have to indicate whether a symptom occurred seldom or never (< 1 day), sometimes or a few times (1–2 days), often (3–4 days), or most of the time/always (5–7 days). Each answer is scored as 0, 1, 2, or 3, respectively, and a total sum score is calculated. The score of the CES-D can thus vary between 0 and 60, with a higher score indicating more depressive feelings. It has been suggested that depression should be considered as a continuum of increasing severity [8]. Therefore, the CES-D score was taken as a continuous variable in the current study. A CES-D score of ≥ 16 is, in general, accepted as an indication of depression [58], although some have suggested that a higher cut-off point might be more appropriate in adolescents, for example, a score of ≥ 22 [59]. We used a score of ≥ 16 in descriptive analyses to indicate the number of adolescents with possible depression in our sample.

#### The Rosenberg Self-Esteem scale

Self-esteem was measured with the Dutch version of the Rosenberg Self-Esteem scale (RSE). The internal reliability for the RSE has been found to be high in adolescents (*α* = 0.88) [60, 61] and construct validity with other measures of self-esteem has been shown [61]. The RSE consists of 10 questions scored on a 4-point response system (from strongly disagree = 0 to strongly agree = 3) requiring participants to indicate their level of agreement with a series of statements about themselves. The total score of the RSE can vary between 0 and 30, with a higher score indicating higher self-esteem. The RSE score was also taken as a continuous variable.

#### Additional measures

Students filled out a questionnaire to assess covariates. The following variables were assessed, and included in the analyses as covariates, as they are known to be associated with depression: BMI (weight/length^2^, self-reported) [62, 63], sex [64, 65], age in years [66], alcohol consumption (number of days per week that alcohol was consumed times units consumed per consumption moment) [67], smoking (yes/no, yes if the participant had indicated to smoke more than 0 cigarettes per week) [68], parental level of education (subdivided into eight levels from primary school to university) [69], pubertal status according to the categorisation suggested by Petersen et al. (subdivided into five levels from prepubertal to postpubertal) [70] (association between pubertal status and depression [71, 72]), and diagnosis which might influence learning (yes/no, e.g. autism, dyslexia, ADHD) [73, 74].

### Data analyses

Twelve participants had a maximum of two missing data points for the CES-D, these missing points were imputed by the person average score of the other CES-D items (if necessary reversed scored) as explained by Bono et al. [75]. For the RSE the same procedure was used. For all continuous variables (all fatty acids, O3I, BMI, CES-D and RSE score) z-scores were calculated, i.e. a measure of how many standard deviations a data point is away from the mean score of the total sample.

We estimated the effect sizes for the association between the fatty acids and depression score/ self-esteem using Bayesian statistical approach. This approach makes it possible to incorporate prior knowledge about relationships (called a prior), and, using a Bayes factor, to compare different models including the model representing the null hypotheses. Bayes factor_10_ (BF) indicates how many times the alternative hypothesis (H_1,_ e.g. DHA is related to depression) is more likely compared to the null hypothesis (H_0,_ e.g. DHA is not related to depression) or another hypothesis. It is, in general, accepted that a BF_10_ between 0.33 and 3 indicates that data do not favour either H_0_ or H_1_, the data are insensitive. A BF_10_ for the comparison of H_1_ to H_0_ of less than 0.33 indicates evidence in favour of the H_0_ and a BF_10_ of more than 3 indicates evidence for the H_1_ [76]. We considered the degree of evidence in favour of a model in accordance with Jeffrey’s classification: BF_10_ of 1 for no evidence; 1 to 3 (resp. when BF_10_ is 1/3 to 1 there is evidence in favour of null hypothesis) for anecdotal evidence in favour of alternative hypothesis; 3–10 (1/10 to 1/3) for substantial evidence; 10–30 (1/30 to 1/10) for strong evidence; 30–100 for very strong evidence, (1/100 to 1/30); > 100 (< 1/100) for extreme evidence [77]. Next to estimating BF_10_ indices, we also used a top-down approach in which the full model is compared with a model with all factors but one (i.e., inclusion of all fatty acids but DHA). BF_omit_ indicates whether removing the factor from the model is deleterious, it is suggested that a BF_omit_ < 3 indicates that the factor is beneficial for the model and a BF_omit_ > 3 that removing the factor is better for the model [78]. Because of the same denominator, BF_10_ indices of different models with all possible combinations of covariates and predictors can be compared. For estimated effect sizes, a 95% credibility interval is provided that describes intervals where the parameters fall within a 95% probability given the observed data.

Analyses were executed in the R statistical environment (R studio version 3.3.2) with the package BayesFactor (version 0.9.12-2). We used the standard settings of the package: the package considers the regression parameters to be distributed normally around zero, with negative effects and positive effects being equally likely, and smaller effects being more likely than larger effects. For the descriptive comparisons of characteristics between those with and without depression, ANOVA and Chi-square analyses were carried out. To explore the associations between each respective fatty acid or the O3I and depression respectively self-esteem, regression models were built predicting CES-D and RSE from the fatty acids. Next to the analyses with each fatty acid as individual predictor, two regression models were additionally built (one for depression and one for self-esteem) with all fatty acids as predictors in one analysis, which allows adjustment of the effect of a fatty acid for the effects of other fatty acids. All regression models were corrected for covariates, which were selected by comparing BFs of regression models with all possible combinations of covariates and the fatty acid. For estimation of the effect size 10.000 Monte Carlo iterations were run.

## Results

In total, 286 students provided informed consent. For three participants no blood sample was obtained. Additionally, one participant was excluded because of severe hyperventilation after blood sampling, one participant withdrew consent before the study started, and one student quit during the first test session and was, therefore, excluded. Fourteen participants had an O3I > 5% and were, therefore, based on the protocol of the main Food2Learn study, excluded. Nine additional students did have an O3I > 5% but were not excluded before the start of the study, they were however excluded from analyses.

In total 257 students started the study; however, baseline data on depression were available for 252 participants and self-esteems scores for 255 participants. Characteristics of all participants are presented in Table 1. Do note that not all participants filled out the personal questionnaire, or did not fill out all questions, and data on level of parental education (LPE) and BMI are missing for 17 participants. A total of 74 (29.4%) students (with CES-D score available) scored ≥ 16 on the CES-D indicative of a depression. When using the stricter criterion of ≥ 22, a total of 43 students (17.1%) could be classified as possibly having depression.

**Table 1:** Participants’ characteristics

	*N*	Mean	SD	Fatty acid (%wt/wt of total FA, except for O3I)	*N*	Mean	SD
Age (years)	257	14.11	0.50	Omega-3 Index	257	3.77	0.55
Male/female	257	124/133 (48.2/51.8%)	–	DHA 22:6n-3	257	2.54	0.46
Smoking no/yes^a^	255	231/24 (89.9/9.3%)	–	EPA 20:5n-3	257	0.38	0.15
BMI	240	19.98	3.00	DPA 22:5n-3	257	1.22	0.19
Alcohol units per week^b^	256	0.47	1.80	AA 20:4n-6	257	11.16	1.27
LPE	240	5.05	1.5	ObA 22:5n-6	257	0.44	0.10

*LPE* level of parental education, *O3I* Omega-3 Index

^a^Smoking was defined as anybody who indicated to smoke more than zero cigarettes per week

^b^Alcohol units per week was operationalized as number of day per week that alcohol is consumed times units per consumption moment

Comparing those with a depression (CES-D ≥ 16) and those without a depression (CES-D ≤ 15), there was extreme evidence (BF_10_ = 1.1E + 4) that girls had more often a depression (42.3% with CES-D ≥ 16) than boys (15.6%). There was substantial evidence (BF = 5.64) that those with a depression had a higher BMI (*M* = 20.84, SD = 3.10) than those without depression (*M* = 19.64, SD = 2.93) and extreme evidence (BF_10_ = 3.7E + 2) that those with a depression were further in puberty (82.9% were advanced or postpubertal) than those without depression (50.9% advanced or postpubertal). There was extreme evidence (BF_10_ = 1.7E + 28) that self-esteem was significantly lower in those with a depression (*M* = 15.4, SD = 5.64) compared to those without (*M* = 23.88, SD = 3.96). Also when looking at the association between self-esteem and depression, their association was strong (self-esteem regressed on depression, regression coefficient = − 0.75 [95% credibility interval − 0.83 to − 0.67]; BF_10_ = 3.51E43).

When comparing models predicting CES-D from potential confounders (namely smoking, alcohol, age, sex, BMI, LPE, pubertal status and diagnosis), the models with the highest BF_10_ included the variables smoking, sex and BMI. We, therefore, included these variables as covariates in the main analyses. For RSE, the best models contained smoking, sex and diagnosis, or smoking and sex. We, therefore, only included smoking, sex and diagnosis as covariates in the analyses for RSE. Excluding participants with missing data, data of 234 participants were available for CESD and data of 253 participants for RSE.

Individual models that predicted CES-D scores from a fatty acid showed negative regression coefficients with 95% credibility intervals without zero for AA [regression coefficient = − 0.14 (95% credibility interval − 0.26 to − 0.03); BF_10_ = 2.37], DPA [− 0.13 (− 0.25 to − 0.02); BF_10_ = 1.08], and ObA [− 0.16 (− 0.28 to − 0.05); BF_10_ = 350.04] (Table 2). This indicates that higher levels of these fatty acids in blood are associated with lower depression scores. However, BF_10_ indicated evidence in favour of the association for ObA only (extreme evidence, BF_10_ > 100). For AA and DPA, BF_10_ showed anecdotal evidence in favour of the alternative hypothesis. The BF_10_ for O3I and DHA were below 0.33, which indicates that there is more evidence for the H_0_, i.e., no association between O3I and DHA, and depression.

**Table 2:** Bayesian analyses, fatty acids as predictor for score on the CES-D, both separate and combined in one model

Predictor fatty acid	One fatty acid				All fatty acids in one model		
	Regression coefficient	95% credibility interval^a^	BF_10_^b^		Regression coefficient	95% credibility interval	BF_omit_^c^
Omega-3 Index	− 0.016	− 0.13;0.10	0.14				
DHA	− 0.04	− 0.16;0.08	0.15	DHA	− 0.02	− 0.14;0.10	3.22
EPA	0.06	− 0.06;0.17	0.37	EPA	0.09	− 0.04;0.21	1.40
AA	− 0.14	− 0.26;− 0.03	2.37	AA	− 0.04	− 0.18;0.11	2.85
DPA	− 0.13	− 0.25;− 0.02	1.08	DPA	− 0.12	− 0.25;0.02	0.83
ObA	− 0.16	− 0.28;− 0.05	350.04	ObA	− 0.10	− 0.22;0.03	1.09

One fatty acid model is a model in which only one predictor of interest is entered (Omega-3 Index, DHA, EPA, AA, DPA, or ObA). All fatty acids model is a model in which all fatty acids (DHA, EPA, AA, DPA, and ObA) are entered, i.e., for example the association between DHA and self-esteem is corrected for the other fatty acids (EPA, AA, DPA, ObA)

All analyses were adjusted for BMI, smoking (yes/no), and sex

*O3I* Omega-3 Index, *CES-D* Centre for Epidemiologic Studies Depression scale

^a^Credibility intervals are analogous to confidence intervals in traditional statistics

^b^BF_10_ refers to the evidence for the model with the specific fatty acid compared to a model with only covariates

^c^BF_omit_ indicates whether the model improves with the omission of that specific fatty acid. BF_omit_ numbers above 3 indicate that keeping the variable in the model is not preferable

When all fatty acids were taken in one model as predictors of CES-D (BF_10_ for the model = 2.4E + 5), the regression coefficient for ObA became smaller and its 95% CI contained a zero score [− 0.10 (− 0.22 to 0.03)] (Table 2). The regression coefficient of DPA was almost identical to that in the individual model − 0.12 (− 0.25 to 0.02), but 95% CI for DPA, as well as for all other fatty acids, contained zero. The BF_omit_ indicated that the model would be improved when DHA was omitted (BF_omit_ > 3). For other acids, BF_omit_ indices were not higher than 3 or lower than 0.33, thus removing them would not improve or worsen the model.

Bayesian analyses for regressions that predicted the RSE score showed no 95% CIs that did not include zero (Table 3). When looking at the Bayes factors, there was only substantial evidence for an association between ObA and RSE (BF_10_ = 4.36), for all other fatty acids, and the O3I, BF_10_ was below 0.33 which indicates more evidence for the H_0_, i.e., no association between fatty acids and self-esteem. When all fatty acids were taken in one model as predictors of RSE, BF_omit_ for DHA exceeded the threshold values (> 3), which indicates that removing this from the model would improve the model that contains all fatty acids.

**Table 3:** Bayesian analyses, fatty acids as predictor for score on the RSE, both separate and combined in one model

Predictor fatty acid	One fatty acid				All fatty acids in one model		
	Regression coefficient	95% credibility interval^a^	BF_10_^b^		Regression coefficient	95% credibility interval	BF_omit_^c^
Omega-3 Index	− 0.05	− 0.16;0.07	0.19				
DHA	− 0.02	− 0.14;0.09	0.15	DHA	− 0.004	− 0.12;0.11	3.28
EPA	− 0.08	− 0. 19;0.04	0.31	EPA	− 0.07	− 0.20;0.05	1.67
AA	− 0.004	− 0.12;0.10	0.14	AA	− 0.06	− 0.20;0.08	2.26
DPA	0.03	− 0.08;0.15	0.17	DPA	0.08	− 0.05;0.21	1.61
ObA	0.09	− 0.03;0.20	4.36	ObA	0.07	− 0.05;0.20	1.54

One fatty acid model is a model in which only one predictor of interest is entered (Omega-3 Index, DHA, EPA AA, DPA or ObA). All fatty acids model is a model in which all fatty acids (DHA, EPA, AA, DPA and ObA) are entered, i.e., for example the association between DHA and self-esteem is corrected for the other fatty acids (EPA, AA, DPA, ObA)

All analyses were adjusted for smoking (yes/no), sex and diagnosis (yes/no)

*RSE* Rosenberg Self-Esteem scale

^a^Credibility intervals are analogous to confidence intervals in traditional statistics

^b^BF_10_ refers to the evidence for the model with the specific fatty acid compared to a model with only covariates

^c^BF_omit_ indicates whether the model improves with the omission of that specific fatty acid. BF _omit_ above 3 indicate that keeping the variable in the model is not preferable

## Discussion

Analyses showed Bayes factors that indicated extreme evidence for a weak negative association between ObA levels and depression score, and substantial evidence for a weak positive association between ObA and self-esteem score. In other words, more ObA measured in blood corresponded with lower depression and higher self-esteem scores. There was no substantial evidence for an association between any of the other fatty acids and depression/self-esteem. For DHA, EPA, and O3I there was even evidence for the absence of an association. When the associations of fatty acids were adjusted for each other’s effects, all effect sizes for tested associations decreased and 95% credible intervals for regression coefficients contained a zero-score. Note that additional non-linear analyses with quadratic fatty acid concentrations (not reported here) did not show evidence for a quadratic effect.

The evidence for the relation of depression score with DHA, EPA and O3I in the current study was in favour of the null hypothesis (i.e., no association). Some earlier studies in adolescents that used the frequentist hypothesis testing showed non-significant associations between fatty acids and depression score [38, 79]. However, the disadvantage of the frequentist hypothesis testing approach is that a non-significant result can either imply that there is evidence for the null hypothesis (i.e., there is no association) or the data are insensitive in distinguishing the theory from the null hypothesis (i.e., nothing follows from the data) [80]. Our findings are in contrast to another earlier study in adolescents from the general population in which an negative association between depression measured with the Beck Depression Inventory (BDI) and EPA in adipose tissue of adolescents was found (i.e., more EPA, lower depression score; analysis was controlled for other fatty acids but no correction for multiple testing) [81]. However, some researchers have suggested that the CES-D, like used in our study, is better at discriminating depression at lower levels than the BDI and the CES-D, thus, might be a better measure of depression in the general population [82, 83]. Furthermore, an association between adipose fatty acids and brain fatty acids has, to our knowledge, not been established, while the association between blood levels and brain levels has been established in animal studies [84]. Blood levels of fatty acids might, therefore, be a better measure for assessing the association between fatty acids and depressive feelings.

It is remarkable that the negative associations of ObA with depression and self-esteem score became less convincing and the relationship between ObA and depression score became weaker after correction for other fatty acids. We are not aware of earlier studies reporting the association between ObA and depression score, although studies looking at other omega-6 fatty acids mostly showed positive associations (i.e., more omega-6 fatty acids, higher depression scores) [85, 86]. ObA can, in the case of DHA deficiency, take the place of DHA in the brain [87]. Possibly the replacement of DHA by ObA does not change the brain functionality and, therefore, a negative association between ObA and depression score in the case of DHA deficiency can be found. This surprising negative association between ObA and depression score does merit more research.

Our data provided evidence for absence of associations of depression score with DHA, EPA and O3I in this adolescent sample, while meta-analyses that included studies in adults have shown that LCPUFA supplementation is associated with less depressive feelings [42, 88, 89]. One of the possible explanations for an absent relationship in the current study could be that the design of this trial led us to preselect participants with an O3I ≤ 5%. In other fatty acid studies, it has been suggested that positive associations with O3I are only visible at higher O3I levels (i.e., 8% and up). For example, in cardiovascular health, an O3I of > 8% is associated with the greatest risk reduction and the study of Markhus et al. showed a linear relation between O3I and depressive symptoms during pregnancy only when the O3I was > 5.1% [50, 52]. Such O3I values seem rare in the specific population of students of LGSE as we only had to exclude 23 out of 286 students for having an O3I > 5%, with the highest O3I being around 6.09%. So, such a low O3I seems to be reality in this specific target group. Furthermore, whenever an effect of omega-3 fatty acids in trials is found it is mostly a small effect, which was also true in this sample where the effect sizes ranged between 0.001 and 0.15. Such a small effect could easily be offset by other environmental factors. This might especially be the case in adolescence, a period of life characterised by profound changes in brain development, but also social and emotional behaviour [90, 91]. Another important issue to consider is that most of the studies looking at depression that find significant associations find very small effect sizes and do not use Bayesian statistics. In the current study, we did find some regression coefficients with 95% CI that did not include 0 (e.g. AA and DPA predicting depression score). ‘Frequentist’ regressions with SPSS software showed comparable regression coefficients that were significant (not shown). However, Bayesian analyses only revealed anecdotal evidence for these associations. This could also be the case for other studies that found significant associations.

This study showed that depressive feelings are common in adolescents of LGSE; in our sample 29.4% of the adolescents scored ≥ 16 on the CES-D which is indicative for a depression. When we used a stricter cut-off point (as some suggest for adolescents) of ≥ 22, 17.1% had scores indicative of depression. Although the percentage of adolescents with depression in this sample seems to be high, it is similar to percentages found in earlier studies in adolescents. Munhoz et al., for example, showed that 17% of the Brazilian adolescents had scores indicative for major depression [92]. Grant and colleagues (2015) also showed numbers comparable to the current study with 22% of the boys and 34.4% of girls showing depressive symptoms [93], and Mamalakis et al. (2016) observed a similar mean CES-D score (mean of 14.9 compared to 12.2 in our sample) [81]. Furthermore, it has long been known that, from age 13–14 years onward, there is a female preponderance of depression [94]. We also showed that girls in this sample had a higher incidence of depression than boys. In the current study, 42.3% of the girls had an indication for depression while, for boys, this was 15.6%.

This observational study has a number of limitations. The low O3I of the participants and low spread in the O3I could have led to low statistical power. However, as we explained above, the low O3I seems to be the reality in this specific target group. Additionally, covariates included in the analysis such as smoking behaviour, drinking behaviour were assessed via self-reports, this could possible lead to bias in these covariates. Also note that the recruitment rate (i.e., percentage of those invited who decided to participate) was approximately 13%, this could limit the external validity. However, such recruitment rate is rather common in a school setting [95]. Moreover, this was a cross-sectional study and can thus not be used for proving causal relationships, experimental studies are needed for more insight in the effects of fatty acids in depression and self-esteem in this specific target group. One of the main strengths of the current study is the use of finger prick blood samples to determine the fatty acids status of the adolescents. Moreover, the population was rather homogenous and this study is one of the first specifically focusing on adolescents of a lower education level. Furthermore, we used the Bayesian approach that, in contrast to the frequentist approach, can be used to provide evidence for the null hypothesis and to compare different models without issues concerning multiple testing.

To summarise, our data in Dutch LGSE adolescents with a low O3I in blood revealed high depression rates and provided extreme evidence for a weak association between ObA levels and depression score (more ObA, lower depression score) and substantial evidence for a weak association between ObA and self-esteem score (more ObA, higher self-esteem). This surprising result calls for more research into the role of ObA in depression. Results of the intervention study Food2Learn should shed light on the effect of krill oil supplementation (high in omega-3 fatty acids) on depressive feelings in adolescents of the LGSE.

## Abbreviations

AA: Arachidonic acid, 20:4n-6
BF: Bayes factor
CES-D: Centre for Epidemiologic Studies Depression scale
DGLA: Dihomo-γ-linolenic acid, C20:3n-6
DHA: Docosahexaenoic acid, C22:6n-3
DPA: Docosapentaenoic acid, 22:5n-3
EPA: Eicosapentaenoic acid, C20:5n-3
LCPUFA: Long-chain polyunsaturated fatty acid
LGSE: Lower general secondary education
O3I: Omega 3-Index
ObA: Osbond acid, n-6 docosapentaenoic acid, 22:5n-6
RSE: Rosenberg Self Esteem Questionnaire
