# Correction to: Exploring the association between whole blood Omega-3 Index, DHA, EPA, DPA, AA and n-6 DPA, and depression and self-esteem in adolescents of lower general secondary education

**DOI:** 10.1007/s00394-019-02175-2

**Published:** 2020-01-10

**Authors:** I. S. M. van der Wurff, C. von Schacky, T. Bergeland, R. Leontjevas, M. P. Zeegers, P. A. Kirschner, R. H. M. de Groot

**Affiliations:** 1 Welten Institute, Research Centre for Learning, Teaching, and Technology, Open University of the Netherlands, Valkenburgerweg 177, P.O. Box 2960, 6419 AT Heerlen, The Netherlands; 2 Omegametrix, 82 152 Martinsried, Germany; 3 Preventive Cardiology, Medical Clinic and Poli-Clinic I, Ludwig Maximilians-University Munich, Munich, Germany; 4 Aker BioMarine Antarctic AS, 1327 Lysaker, Norway; 5 Faculty of Psychology and Educational Sciences, Open University of the Netherlands, 6419 AT Heerlen, The Netherlands; 6 Nutrition and Translational Research in Metabolism (School NUTRIM), Maastricht University, 6200 MD Maastricht, The Netherlands; 7 Care and Public Health Research Institute (School CAPHRI), Maastricht University, 6200 MD Maastricht, The Netherlands; 8 University of Oulu, Oulu, Finland

## Correction to: European Journal of Nutrition (2019) 58:1429–1439 10.1007/s00394-018-1667-4

The original version of this article unfortunately contained a mistake. Title was incorrect.

The correct title should be:

Exploring the association between whole blood Omega-3 Index, DHA, EPA, DPA, AA and n-6 DPA, and depression and self-esteem in adolescents of lower general secondary education

